# Modelling electron-phonon interactions in graphene with curved space hydrodynamics

**DOI:** 10.1038/s41598-018-30354-4

**Published:** 2018-08-22

**Authors:** Ilario Giordanelli, Miller Mendoza, Hans Jürgen Herrmann

**Affiliations:** 1 ETH Zürich, Computational Physics for Engineering Materials, Institute for Building Materials, Wolfgang-Pauli-Strasse 27, 8093 Zürich, Switzerland; 2 Universidade Federal do Ceará, Departamento de Física, Campus do Pici, 60455-760 Fortaleza, Ceará, Brazil

## Abstract

We introduce a different perspective describing electron-phonon interactions in graphene based on curved space hydrodynamics. Interactions of phonons with charge carriers increase the electrical resistivity of the material. Our approach captures the lattice vibrations as curvature changes in the space through which electrons move following hydrodynamic equations. In this picture, inertial corrections to the electronic flow arise naturally effectively producing electron-phonon interactions. The strength of the interaction is controlled by a coupling constant, which is temperature independent. We apply this model to graphene and recover satisfactorily the linear scaling law for the resistivity that is expected at high temperatures. Our findings open up a new perspective of treating electron-phonon interactions in graphene, and also in other materials where electrons can be described by the Fermi liquid theory.

## Introduction

At finite temperatures, phonons interact with charge carriers, and therefore, contribute to the electrical resistivity of the respective material^1^. For most solids, the temperature behaviour of the electrical resistivity changes at the Debye temperature, which corresponds to the theoretical highest phonon frequency in the material. In graphene, the Debye temperature is very high, $T\approx 2300$ K and $T\approx 1300$ K for planar and out-of-plane phonons, respectively^2^. However, for low electron densities, the Fermi energy can be substantially smaller than the Debye energy, and only phonons with energy smaller than two times the Fermi energy, corresponding to a full backscattering of electrons, can scatter with the electrons. This defines the Bloch-Grüneisen temperature *T*_*BG*_^3^, which for doped suspended graphene is around 100 K^4,5^. Therefore, in suspended graphene, the behaviour of the resistivity changes at Bloch-Grüneisen temperature ${T}_{BG}$. It has been observed, that the temperature dependence of the resistivity follows a linear relation due to electron-phonon interactions above 100K and even becomes quadratic in the absence of any external strain^6,7^

Recently it has been shown that the electronic flow in graphene can be modelled using hydrodynamic equations^8–16^. Hydrodynamics is an effective theory that describes the relaxation of any interacting system to thermal equilibrium, where the disorder only varies on long wavelengths compared to the electron-electron scattering length^11,17,18^. Several different models are used to account for the electron-phonon interaction in the hydrodynamic picture. Usually, in this hydrodynamic formalism, electron-phonon interactions are included into the equation for the electrical current density $\overrightarrow{J}$ as a damping term, $-\overrightarrow{J}/{\tau }_{p}$, with a characteristic relaxation time *τ*_*p*_^15,16^. More elaborate theories model the presence of internal strain on a crystal lattice as an effective distortion to the induced metric^19,20^. Here, we present an approach based on the induced metric, where we account for the electron-phonon interactions by including inertial corrections due to the deformations of the graphene sheet. The novelty in our approach relies on the coupling of the hydrodynamics electron fluid with the atomistic simulation of graphene sheets. The deformations used in our model are directly derived from atomistic simulations. The graphene sheet in this atomistic simulation behaves as a two-dimensional manifold that changes in time due to thermal fluctuations. The electrons flow through this manifold when an external electric field is applied. In order to describe the electrons with hydrodynamic equations, we use a covariant representation where the existence of deformations translates into the appearance of inertial corrections. In this work, we considered doped graphene samples, i.e. we are away from the charge neutrality point, where charge carriers are induced by a gate voltage instead of thermal fluctuations, and therefore the Fermi liquid theory applies^21^.

Other models have been used to describe electron-phonon interactions as inertial forces^22,23^. However, in this work, we use an approach in which the thermal fluctuations are responsible for inducing curvature in the graphene sheets. This curvature generates dissipation^24^ and electrical resistivity in the sample. The resulting inertial terms are added naturally to the electronic fluid equations depending on the complexity of the locally induced curvature. To the best of our knowledge, this approach has never been proposed before. With our model at hand, we address the question whether the curvature induced inertial corrections can recover the temperature relation of the electrical resistivity in the high temperature regime.

## Method

**Figure 1:**
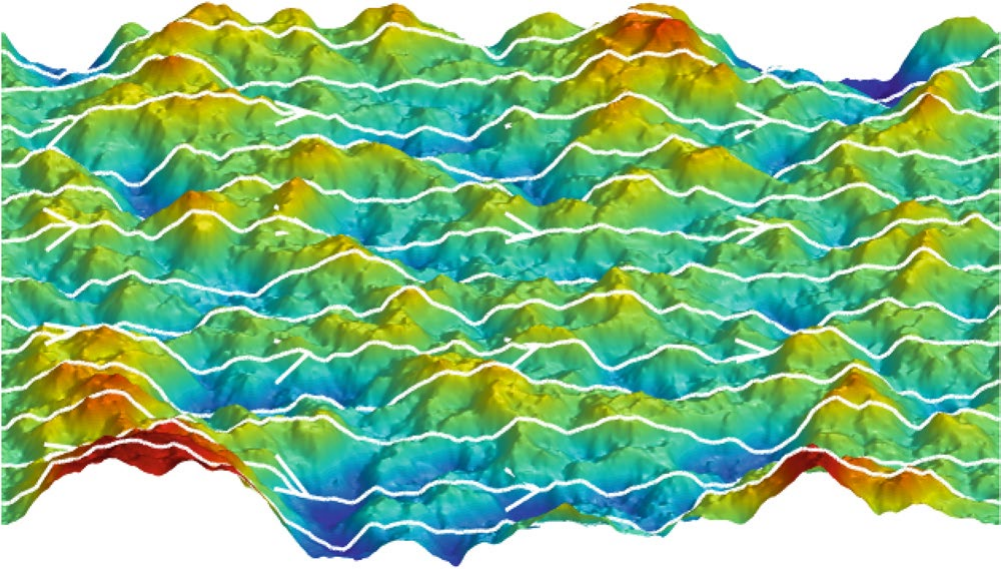
Graphene membrane at 300 K after the electronic flow has reached a steady state. Streamlines indicate the velocity field of the electronic flow that moves from left to right. The colours represent the height.

We use molecular dynamics simulations to simulate different graphene membranes at different temperatures and imposed strains. From the position of the atoms, we build the coordinate system in which the electrons flow, and extract the inertial corrections to include them into the two-dimensional hydrodynamic equations. In Fig. 1, we can observe how the presence of thermal fluctuations produces curved streamlines of the electron velocity field.

**Figure 2:**
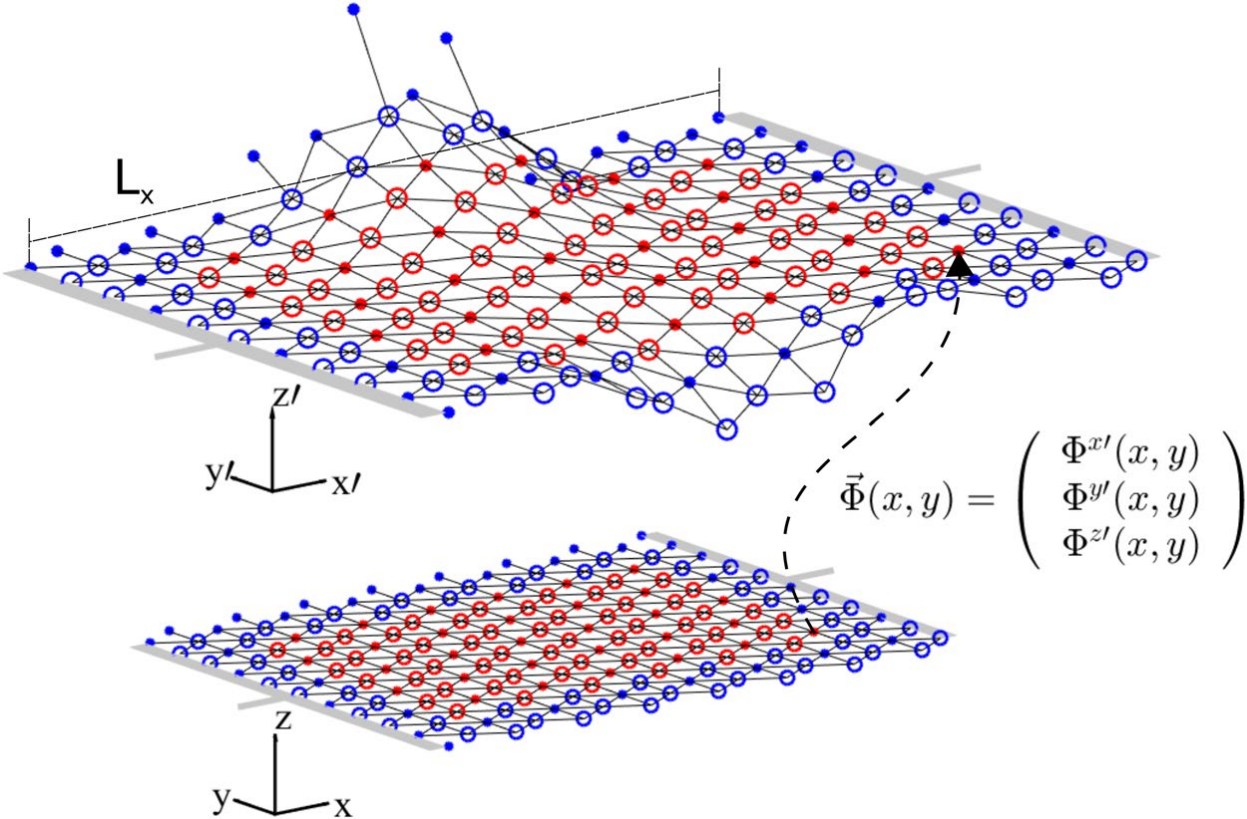
Discrete mapping (dashed arrow) that describes the embedding of the two-dimensional triangular lattice (bottom figure) in the three-dimensional Cartesian space (top figure). The circles represent the atoms. The electrical contacts are shown in grey at both ends of the graphene sheet.

To simulate the graphene sheets with molecular dynamics we use the adaptive intermolecular reactive bond-order (AIREBO) potential^25^. This many-body potential has been developed to simulate molecules made of carbon and hydrogen atoms, and has been very successful to reproduce the phonon dispersion curves of graphene^26^. In our simulations we consider suspended graphene sheets of *L*_*x*_ = 99.2 Å, (see Fig. 2), but also two stretched cases with 100.6 Å and 102.1 Å. As shown in Fig. 2 the zigzag edge is located along the *x*-direction (top and bottom) and accordingly the armchair edge along the *y*-direction (left and right). The atoms can freely move in all directions, except the ones at the left and right boundary, representing the electrical contacts (grey in Fig. 2). The distance between contacts will be denoted by *L*_*x*_. Note that since the left and right boundaries are fixed, we are effectively applying strain to the graphene samples, and therefore, expect a linear dependence of the resistivity with temperature. We simulate several samples at different temperatures within the range of 100–600 K controlling the temperature of the system with a Nosé-Hoover thermostat. All molecular dynamics simulations run (during the thermalisation procedure) with a time step Δ*t* = 1 fs, which is small enough to capture the dynamics of the carbon-carbon interactions accurately. Randomised velocities are attributed to the atoms at the beginning of each simulation. After reaching thermal equilibrium we are ready to couple the graphene sheet to the fluid solver and introduce electrical currents.

The electronic flow in flat graphene can be modelled using hydrodynamic equations, which are responsible for the conservation of particles, ${\partial }_{\mu }{N}^{\mu }=0$, and energy and momentum, ${\partial }_{\mu }{T}_{0}^{\mu \nu }=0$, where *N*^*μ*^ and *T*_0_ are the 3-particle flow and the energy-momentum tensor, respectively. The energy momentum tensor in Minkowski-space *T*_0_ depends on the Minkowski metric *η*_*μν*_ and the shear-stress tensor *π*^*μν*^, which contains the shear viscosity *κ*. The equation of state completes this set of equations, which for the case of graphene is given by *ε* = 2*p*, where *ε* is the energy density and *p* the pressure. To take into account the curvature of suspended graphene sheets, we consider inertial corrections in these equations. For this purpose, we first use the covariant formulation of the hydrodynamic equations in curved space to determine the terms responsible for the inertial corrections, and afterwards, we introduce them as a forcing term into the respective equations for flat graphene. The conservation equations in curved manifolds are derived by replacing partial derivatives ∂_*μ*_ by covariant derivatives ▽_*μ*_ and considering the energy-momentum tensor in curved space *T*^*μν*^, which depends on the generalised metric *g*_*μν*_ instead of the Minkowski (flat) metric *η*_*μν*_. The covariant derivative ▽_*μ*_ in the curvilinear coordinate system can be expressed through the respective partial derivative by ${\nabla }_{\mu }{N}^{\mu }={\partial }_{\mu }{N}^{\mu }+{{\rm{\Gamma }}}_{\mu \lambda }^{\mu }{N}^{\lambda }$, and ${\nabla }_{\mu }{T}^{\mu \nu }={\partial }_{\mu }{T}^{\mu \nu }+{{\rm{\Gamma }}}_{\mu \lambda }^{\mu }{T}^{\lambda \nu }+{{\rm{\Gamma }}}_{\mu \lambda }^{\nu }{T}^{\mu \lambda }$, with ${{\rm{\Gamma }}}_{\mu \lambda }^{\mu }$ being the Christoffel symbols, which are responsible for inertial corrections. With the help of the previous equations we can rewrite the inertial forces as

1a $${F}_{N}=-{{\rm{\Gamma }}}_{\mu \lambda }^{\mu }{N}^{\lambda }$$

1b $${F}_{T}^{\nu }=-\,{{\rm{\Gamma }}}_{\mu \lambda }^{\mu }{T}_{0}^{\lambda \nu }-{{\rm{\Gamma }}}_{\mu \lambda }^{\nu }{T}_{0}^{\mu \lambda }-({\eta }^{\mu \nu }-{g}^{\mu \nu }){\partial }_{\mu }p,$$

for the conservation of particles and energy-momentum, respectively. For more details on hydrodynamics in curved manifolds, see ref^27^.

At this stage we include a temperature independent coupling constant *α* in front of the inertial corrections which accounts for the strength of the electron-phonon interaction. Thus, the set of equations can be written as

2a $${\partial }_{\mu }{N}^{\mu }=\alpha {F}_{N},$$

2b $${\partial }_{\mu }{T}_{0}^{\mu \nu }=\alpha {F}_{T}^{\nu }\mathrm{.}$$

To solve these equations, we make use of the lattice Boltzmann solver described in refs^14,28^, and add the inertial contributions as external forces (see Additional Information for technical details on the numerical implementation). Our model discretises space as regular triangular lattice, which we couple to graphene’s hexagonal lattice. As shown in Fig. 2, the atoms form a two-dimensional manifold which can be described by a discrete mapping ${\rm{\Phi }}(ct,x,y)$ from the curved space to the three-dimensional flat space (reference frame of the laboratory, where the metric is given by the Minkowski-metric). The metric tensor can be computed by

3 $${g}_{\mu \nu }=\frac{\partial {{\rm{\Phi }}}^{\alpha }(ct,x,y)}{\partial {x}^{\mu }}\frac{\partial {{\rm{\Phi }}}^{\beta }(ct,x,y)}{\partial {x}^{\nu }}{\eta }_{\alpha \beta }\mathrm{.}$$

The graphene sheet possesses zigzag boundaries at the left and right end. For the fluid solver we impose periodic boundary conditions at these boundaries, which correspond to the in- and outlet. The free sides of the graphene sheet possesses armchair geometry and, therefore, we impose free slip boundary conditions.

We perform simulations in the Fermi liquid regime, in which the Fermi energy fixes the total number of charge carriers, and can determine whether electrons or holes dominate the charge carriers and electrical currents. Therefore, we set a constant particle density *n* = 1.36 × 10^13^ /cm ^2^ and compare our results with the experimental set-up proposed by Efetov *et al*.^4^. We impose a constant particle density by coupling the fluid solver to a particle (charge) reservoir, thus fulfilling overall particle number conservation even for *α* ≠ 1. Therefore, at every time step during our simulations, we compensate the loss/gain of particles by injecting/subtracting charge carriers, such that the total number of particles is always conserved. This fulfills the role of the gate voltage to fix the Fermi level and therefore, the total number of carriers. To produce an electrical current, we apply an external electric field in *x* direction, *E*^*x*^ = 1.43 × 10^9^ − 10^11^ V/m. In our simulations, we also set the chemical potential $\mu =\hslash {v}_{F}\sqrt{\pi n}\approx 6.89\times {10}^{-20}$ kgm^2^/s^2^$\,\approx \,0.43$ eV, and $p=\frac{1}{3}\mu n\approx 3.12\times {10}^{-3}$ kg/s^2^. We use the shear viscosity for doped graphene given by^10^

4 $$\kappa (T)={c}_{\nu }n{(\frac{\mu }{{k}_{B}T})}^{\frac{3}{2}},$$

with ${c}_{\nu }\approx 1.33\times {10}^{-34}{{\rm{kgm}}}^{2}/s\approx \frac{5}{4}\hslash$. We couple the electronic fluid to the atomistic simulation using the same length-scale *x*_0_ = Δ*x*_*MD*_ = 1 Å and time scale *t*_0_ = Δ*t*_*MD*_ = 10^−5^ fs. After each iteration we compute the metric tensor and the Christoffel symbols and simulate the electronic flow until we obtain the electrical current at steady state. Note that since the electronic flow is much faster than the typical velocity of the carbon atoms, the electrons will reach stationary solutions under adiabatic approximations. However, we still couple both time and space variations on an equal footing. The momentum change in the fluid is imposed on the atoms to ensure momentum conservation of the total system even for $\alpha \ne 1$ in Eq. (2).

Our numerical solver can reproduce both the non-relativistic and relativistic regimes of the hydrodynamics equations, as has been shown in ref^13^. Therefore, we can use it to simulate the electronic flow in doped graphene samples (away from the charge neutrality point) within the Fermi liquid theory.

## Results

**Figure 3:**
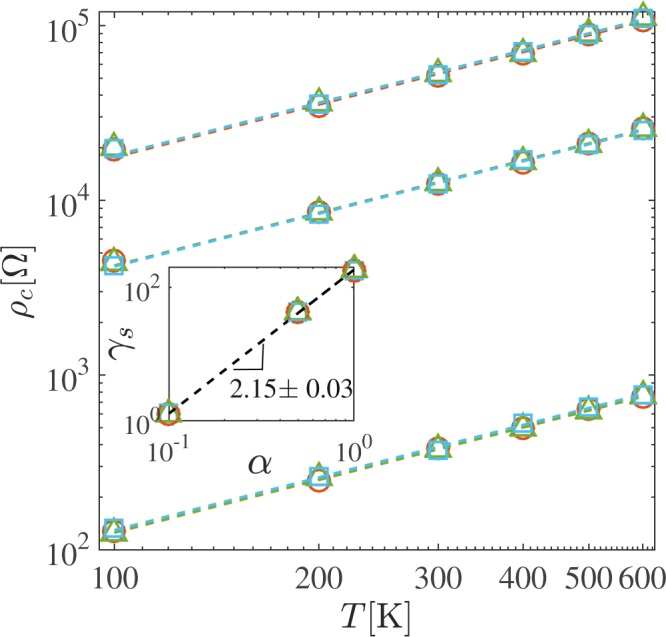
Temperature dependence of the electrical resistivity of graphene due to inertial corrections for different coupling constants *α*. The dashed lines correspond to the best fit for the function *ρ* = *γ*_*s*_*T* + *ρ*_0_ = *ρ*_*c*_ + *ρ*_0_ for *α* = {1.0, 0.5, 0.1} with *γ*_*s*_(*α* = 1.0) = 180 ± 20 Ω/K,*γ*_*s*_(*α* = 0.5) = 44 ± 7 Ω/K and *γ*_*s*_(*α* = 0.1) = 1.3 ± 0.1 Ω/K. The colors represent graphene membranes with different strains where red circles stand for a graphene sheet with size *L*_*x*_ = 99.2 Å, green triangles for *L*_*x*_ = 100.6 Å and blue squares for *L*_*x*_ = 102.1 Å. Inset: Log-log plot of *γ*_*s*_ dependence on *α*. In both figures, the errorbars are smaller than the symbols.

We perform simulations for different strains and different values of the coupling constant and measure the resistivity of the graphene sheets using Ohm’s law, $\langle \rho \rangle ={E}^{x}{L}_{y}/I(t\to \infty )$, where the electrical current is given by $I(t)={\int }_{0}^{{L}_{y}}en(ct,x={L}_{x},y){U}^{x}(ct,x={L}_{x},y)dy\mathrm{.}$ In Fig. 3, we observe that all our calculations of the electrical resistivity of graphene exhibit a linear dependence with temperature,

5 $$\rho ={\gamma }_{s}(\alpha )T+{\rho }_{0}(\alpha ,{L}_{x}\mathrm{).}$$

This linear dependence is in agreement with experimental measurements and theoretical predictions^4,5^. Additionally, the slope *γ*_*s*_(*α*) only depends on the strength of the coupling constant *α* but not on the applied strain, which is also in agreement with previous works^7^. We can also observe from the inset of Fig. 3 that *γ*_*s*_∝*α*^2.15 ± 0.03^. The factor *γ*_*s*_ can be compared with the values from the Boltzmann transport theory^5^, where

6 $$\rho =\gamma T\equiv \frac{\pi {D}^{2}{k}_{B}}{4{e}^{2}\hslash {\rho }_{m}{v}_{ph}^{2}{v}_{F}^{2}}T,$$

with *ρ*_*m*_ = 7.6×10^−7^ kg/m^2^ being the mass density of graphene, *v*_*ph*_ the phonon velocity and *D* the deformation-potential coupling constant. Experimental and theoretical results suggest that the deformation-potential varies within the range of $10\le D\le 20$ eV^6^, and phonon velocities within $2\times {10}^{4}\le {v}_{ph}\le 3\times {10}^{4}$ m/s^29–31^. Thus, one expects *γ* to be in the range $0.06\le \gamma \le \mathrm{0.45\ }{\rm{\Omega }}$/K. From our results we deduce that $0.024\le \alpha \le 0.061$ to obtain results compatible with *γ*. The residual resistivity *ρ*_0_ is 704 ± 33 Ω for *α* = 0.1 and is a consequence of the static corrugations (ripples) present at any temperature. These ripples stabilize the two-dimensional crystal circumventing the Mermin Wagner theorem, which states that crystalline order cannot exist in two dimensions^32^.

The linear temperature dependence observed in this set-up is related to the presence of external strain imposed by fixing the contact points at the boundary. For the study of the strain free resistivity, we have performed molecular dynamics simulations of graphene sheets without restricting the motion of the carbon atoms at the left and right boundary. We have observed that in this case, the linear behaviour of the resistivity is not recovered. Instead, in agreement with refs^6,7^, the data suggest a quadratic dependence of the resistivity on the temperature (see Fig. 1 of the Supplementary Information).

**Figure 4:**
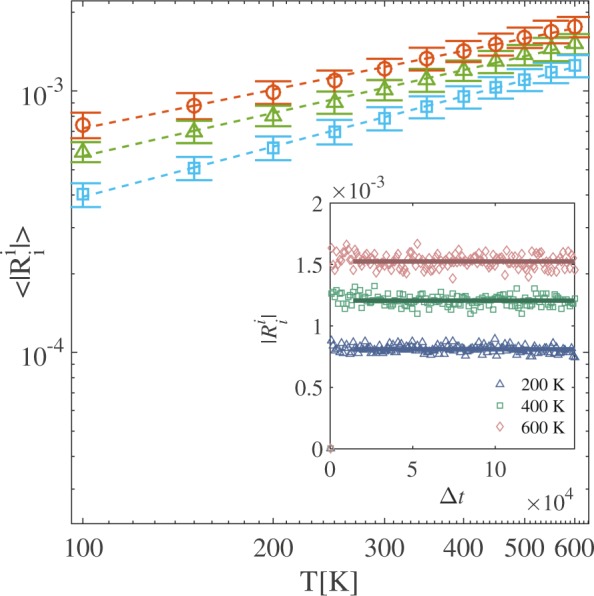
Maximal absolute value of the Ricci scalar $|{R}_{i}^{i}|$ for graphene membranes with distances *L*_*x*_. Main panel: time averaged Ricci scalar $\langle |{R}_{i}^{i}|\rangle$ for different temperatures and distances *L*_*x*_. Red circles correspond to *L*_*x*_ = 99.2 Å, with an exponent 0.49 ± 0.02. Green triangles correspond to *L*_*x*_ = 100.6 Å, with an exponent 0.55 ± 0.03. Blue squares correspond to *L*_*x*_ = 102.1 Å, with an exponent 0.64 ± 0.01. Inset panel: Time evolution of $|{R}_{i}^{i}|$ for graphene membranes at different temperatures for *L*_*x*_ = 102.1 Å.

The existence of a resistivity points to the presence of dissipation. Recently, it has been shown that energy dissipation in flows through curved manifolds arises from the curvature^24^. The Ricci scalar (or curvature scalar) is a measure for the curvature and is given by ${R}_{\mu }^{\mu }={g}^{\nu \sigma }({{\rm{\Gamma }}}_{\nu \sigma ,\mu }^{\mu }-{{\rm{\Gamma }}}_{\nu \mu ,\sigma }^{\mu }+{{\rm{\Gamma }}}_{\nu \sigma }^{\delta }{{\rm{\Gamma }}}_{\mu \delta }^{\mu }+{{\rm{\Gamma }}}_{\nu \mu }^{\delta }{{\rm{\Gamma }}}_{\sigma \delta }^{\mu })$. We have analysed the Ricci scalar for different strains and temperatures and found that its maximal absolute value remains constant during time evolution and depends only on the temperature of the system (see inset of Fig. 4). As function of temperature the Ricci scalar follows a power-law with an exponent that increases with the applied strain (see main panel in Fig. 4).

We have also studied the standard deviation of the heights of our graphene sheets (which represents out-of-plane vibrations), and found that they are virtually not influenced by temperature, within the range of 100–600 K (see Fig. 2 of Supplementary Information). However, we observe that temperature does affect the bond-length distribution, as seen in Fig. 3a–c in the Supplementary Information, meaning that it influences the in-plane vibrations of the membranes. Thus, one can conclude that the linear dependence of the resistivity with temperature is mainly due to in-plane phonons, and may be related to the lack of out-of-plane vibrations, which are suppressed when an external strain is applied (see inset of Fig. 2 in the Supplementary Information). This also explains why in the absence of strain, i.e. without the suppression of out-of-plane vibrations, the quadratic behaviour is recovered.

## Conclusion

To summarise, we have shown that inertial corrections lead to a linear dependence of the electrical resistivity with temperature in suspended graphene under applied strain, which is in agreement with experimental measurements of the electrical resistivity due to electron-phonon interactions at high temperatures. To characterize the strength of the electron-phonon interaction in our model, we have introduced a coupling constant *α*, and for values of $\alpha \approx 0.05$ one expects to recover the same values of *γ* known from theoretical predictions and experimental measurements. Our measured resistivity does not depend on the strength of the strain applied to the sample, however, in the complete absence of external strain it exhibits the expected quadratic temperature dependence^6,7^.

We also analysed the maximal absolute value of the curvature as function of temperature and found a power-law behaviour with an exponent that changes slightly with the applied strain. Finally, by studying the average height fluctuations of the graphene sheets under strain, we also discovered that the height fluctuations are almost temperature independent, and consequently, they seem to be uncorrelated with the changes in curvature. In contrary, the bond-length distribution (which is related to the in-plane phonons) possesses a clear temperature dependence and accounts for larger local values of curvature when increasing the temperature. In our model, larger curvature implies more energy dissipation, and consequently, larger electrical resistivity.

Our finding opens up many interesting questions, as for instance, if one can discern the influence of different types of vibrational phonons, e.g. flexural, acoustic, and optical, by studying how they introduce curvature into the hydrodynamic system. By extending our molecular dynamics simulations, one could also explore the electrical resistivity due to the electron-phonon interactions when the graphene sample is placed on a substrate^30^.

In our work, the adiabatic motion of the electronic flow (weak MD-hydro coupling) relies mainly on two facts: i) our approach is classical, and ii) the electronic flow moves much faster than the carbon atoms. Therefore, the electrical current reaches convergence very fast and one does not need to take into account the simultaneous motion of the graphene structure. This leads us to conclude that within a classical picture of the electron-phonon interaction in graphene, one can consider the spatial static curvature of the sample to explain the resistivity vs. temperature curves found experimentally in graphene. However, other features of this interaction coming from quantum effects are obviously not covered.

Finally, our approach can also be applied to other two- and three-dimensional crystals, where electrons are well described as Fermi liquid. In those structures, ion displacements due to phonons can induce intrinsic curvature, and consequently, energy dissipation in the electronic flow. Applying our model to other materials will be an interesting subject for future research.

## Electronic supplementary material

Supplementary Material
