# Supplementary material for: Modelling electron-phonon interactions in graphene with curved space hydrodynamics
Source: Sci Rep. 2018 Aug 22;8:12545. doi: 10.1038/s41598-018-30354-4 (PMC6105604; doi:10.1038/s41598-018-30354-4)
Supplement: Supplementary file 1 — Supplementary Material [file 41598_2018_30354_MOESM1_ESM.pdf]

Supplementary Material: Modelling electron-phonon interactions in graphene with curved space hydrodynamics

I.Giordanelli[1,*], M. Mendoza[1], and H. J. Herrmann[1,2]

[1]ETH Zürich, Computational Physics for Engineering Materials, Institute for Building Materials, Wolfgang-Pauli-Strasse 27, HIT, CH-8093 Zürich, Switzerland
[2]Universidade Federal do Ceará, Departamento de Física, Campus do Pici, 60455-760 Fortaleza, Ceará, Brazil
[*]gilario@ethz.ch

ABSTRACT

In this Supplementary Material we provide additional information on the conservation equations for curved spaces. We also describe in full length the dimensionless quantities used for the lattice Boltzmann simulations. Additionally, we simulate graphene sheets in the absence of external strain and measure the resistivity. We find that the data suggest a quadratic temperature dependence in the strain-free case. Finally, we study the bond length distribution of the carbon atoms for different external strains.

Conservation equations for curved spaces

The lattice Boltzmann model for flat space reported in Ref.[1] needs to be extended in order to simulate relativistic hydrodynamics in curved spaces. This numerical model solves the following conservation equations:

$$\partial_\mu N^\mu = 0, \tag{1a}$$

$$\partial_\mu T_0^{\mu\nu} = 0, \tag{1b}$$

where $T_0^{\mu\nu}$ is the energy momentum tensor for flat space,

$$T_0^{\mu\nu} = (\varepsilon + p)\frac{U^\mu U^\nu}{v_F^2} - p\eta^{\mu\nu} + \pi^{\mu\nu}. \tag{2}$$

In curved spaces, the energy momentum tensor has to be conserved as well. However, conservation of quantities in a curved manifold needs to be expressed through covariant derivatives, instead of partial derivatives. The conservation equations for the number of particles and energy momentum tensor are given by

$$\nabla_\mu N^\mu = 0, \tag{3a}$$

$$\nabla_\mu T^{\mu\nu} = 0, \tag{3b}$$

where the covariant derivatives ∇_μ of the 3-particle flow and energy-momentum tensor relate to the partial derivatives ∂_μ as follows:

$$\nabla_\mu N^\mu = \partial_\mu N^\mu + \Gamma^\mu_{\mu\lambda} N^\lambda, \tag{4a}$$

$$\nabla_\mu T^{\mu\nu} = \partial_\mu T^{\mu\nu} + \Gamma^\mu_{\mu\lambda} T^{\lambda\nu} + \Gamma^\nu_{\mu\lambda} T^{\mu\lambda}, \tag{4b}$$

where $\Gamma^\mu_{\mu\lambda}$ denote the Christoffel symbols which are computed with the metric tensor using

$$\Gamma^\sigma_{\mu\nu} = \frac{1}{2} g^{\sigma\rho}\left(\frac{\partial g_{\rho\mu}}{\partial x^\nu} + \frac{\partial g_{\rho\nu}}{\partial x^\mu} - \frac{\partial g_{\mu\nu}}{\partial x^\rho}\right) \quad . \tag{5}$$

Additionally, the energy momentum tensor in curved manifolds reads

$$T^{\mu\nu} = (\varepsilon + p)\frac{U^\mu U^\nu}{v_F^2} - p g^{\mu\nu} + \pi^{\mu\nu}, \tag{6}$$

where $g^{\mu\nu}$ is the metric tensor and $\pi^{\mu\nu}$ the shear-stress tensor, which can be approximated by the equation $\pi^{\mu\nu} \approx \kappa(g^{\mu\lambda}\nabla_\lambda U^\nu + g^{\nu\lambda}\nabla_\lambda U^\mu)$. Using the property that $\nabla_\mu g^{\mu\nu} = 0$, we can rewrite the covariant derivative of the energy-momentum tensor as

$$\nabla_\mu T^{\mu\nu} = \nabla_\mu T_0^{\mu\nu} + (\eta^{\mu\nu} - g^{\mu\nu})\nabla_\mu p. \tag{7}$$

Thus the conservation equations (3) expressed with the energy-momentum tensor for the flat space $T_0^{\mu\nu}$ read

$$\begin{aligned} 0 = \nabla_\mu T^{\mu\nu} &= \nabla_\mu T_0^{\mu\nu} + (\eta^{\mu\nu} - g^{\mu\nu})\nabla_\mu p \\ &= \partial_\mu T_0^{\mu\nu} + \Gamma^\mu_{\mu\lambda} T_0^{\lambda\nu} + \Gamma^\nu_{\mu\lambda} T_0^{\mu\lambda} + (\eta^{\mu\nu} - g^{\mu\nu})\partial_\mu p, \end{aligned} \tag{8}$$

where we have used the fact that partial derivatives equal covariant derivatives when acting on scalars. At this stage we also include a temperature independent coupling constant α which accounts for the strength of the inertial corrections. Thus, this set of equations can be written as

$$\partial_\mu N^\mu = \alpha F_N, \tag{9a}$$

$$\partial_\mu T_0^{\mu\nu} = \alpha F_T^\nu, \tag{9b}$$

with

$$F_N = -\Gamma^\mu_{\mu\lambda} N^\lambda \tag{10a}$$

$$F_T^\nu = -\Gamma^\mu_{\mu\lambda} T_0^{\lambda\nu} - \Gamma^\nu_{\mu\lambda} T_0^{\mu\lambda} - (\eta^{\mu\nu} - g^{\mu\nu})\partial_\mu p. \tag{10b}$$

Here F_T^ν and F_N can be introduced in the numerical model as external forces using the technique described in Ref.[2]

Dimensionless quantities

In order to obtain general results we transform the hydrodynamic equations to dimensionless form and determine the dimensionless quantities that characterise our systems. We also include a force density $\vec{F}^{ext} = ne\vec{E}$ describing an external electric field $\vec{E}$ to the energy-momentum conservation equation (**??**).

To write the equations in a dimensionless form, we first express all relevant quantities in dimensionless form: $x = x_0 x', u = u_0 u', t = t_0 t', \varepsilon = \varepsilon_0 \varepsilon', E = E_0 E'$, $\kappa = \kappa_0 \kappa'$ $e = e' e_0$, $n = n'/x_0^2$ and $v_F = u_0 v_F'$ where all primed variables are dimensionless. Furthermore, the relation $u_0 t_0 = x_0$ holds, which allows us to write the temporal derivative $\partial_0 = (1/v_F)\partial_t = (1/v_F' u_0 t_0)\partial_{t'} = (1/v_F' x_0)\partial_0'$, and thus for $v_F' = 1$, the relation $\partial_\mu = (1/x_0)\partial_\mu'$ is also satisfied.

We divide the energy-momentum tensor in an equilibrium part and a dissipative part $T^{\mu\nu} = T^{\mu\nu(eq)} + \pi^{\mu\nu}$. The equilibrium part can be written in its dimensionless form $T^{\mu\nu(eq)} = \varepsilon_0 T'^{\mu\nu(eq)}$. Assuming that the shear-stress tensor is linearly dependent on the shear viscosity κ [3, p. 109] and taking into account that the shear stress tensor has the same units as the energy-momentum tensor ($\kappa_0 = \frac{\varepsilon_0 x_0}{u_0}$), we get $\pi^{\mu\nu} = \kappa_0(\varepsilon_0/\kappa_0)\pi'^{\mu\nu} = \kappa_0(u_0/x_0)\pi'^{\mu\nu}$. The force densities (10) transform as $F_N = (\varepsilon_0/x_0)F_N'$ and $F_T^\nu = (\varepsilon_0/x_0)F_T'^\nu$, respectively. This can be derived from the fact that, in our case, the metric is dimensionless and thus the Christoffel symbols (as derivative of the metric) transform as $\Gamma^{\mu\nu}_\sigma = (1/x_0)\Gamma'^{\mu\nu}_\sigma$.

With the considerations above, equations (**??**) read

$$\frac{1}{x_0^3}\partial_\mu' N'^\mu = \frac{1}{x_0^3}F_N', \tag{11a}$$

$$\frac{\varepsilon_0}{x_0}\partial_\mu' T'^{\mu\nu(eq)} + \frac{\kappa_0 u_0}{x_0^2}\partial_\mu' \pi'^{\mu\nu(eq)} = \frac{\varepsilon_0}{x_0}F_T'^\nu + \frac{e_0 E_0}{x_0^2} n' e' E'^\nu. \tag{11b}$$

While the first equation remains unchanged, we observe after multiplying by (x_0/ε_0) that the second equation only depends on two dimensionless numbers

$$A_1 = \frac{\kappa_0 u_0}{x_0 \varepsilon_0} \text{ and } A_2 = \frac{e_0 E_0}{x_0 \varepsilon_0}. \tag{12}$$

Note that $1/A_1$ is related to the Reynolds number.[4] The parameter range of our simulations is $0.14 \le A_1 \le 2.05$ and $10^{-8} \le A_2 \le 10^{-6}$.

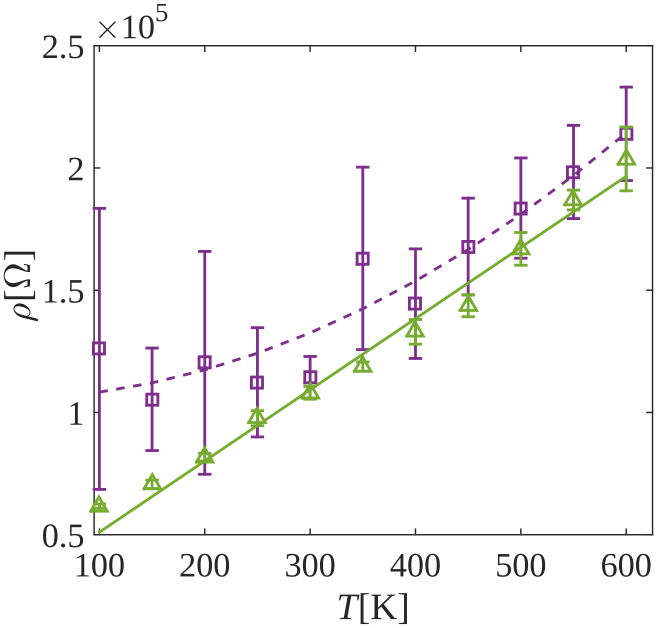

Figure 1. Temperature dependence of the electrical resistivity of graphene. The violet squares represents simulation results performed in complete absence of any external strain (free boundary molecular dynamics simulations, without restricting the motion of the atoms at the left and right boundary). The violet dashed line correspond to the best fit for the function $\rho = \gamma_{free} T^2 + \rho_0$. For comparison, we have also included the data of the resistivity from simulations with restriction on the left and right boundary. This data is denoted by green triangles and, in that case, the graphene has a total size $L_x = 100.6$ Å. The green line represents the best fit of this data with a linear function.

Resistivity in the absence of external induced strain

The simulations of the suspended graphene samples have all non-zero strain due to the way we introduce the contact points at the left and right boundaries, i.e. the carbon atoms located at those positions are forced to remain immovable. In order to study the case of no strain we have performed simulations of graphene membranes without restricting the motion of the atoms at the boundaries. As shown in Fig. 1, in the absence of any external induced strain we observe that the linear temperature dependence of the resistivity is not recovered. Instead, the data suggest a quadratic dependence of the resistivity on the temperature, in agreement with Refs.[5,6]

Strain induced by the contact points

All simulations are performed with the same number of carbon atoms ($N = 4114$). The atoms at the left and right boundary represent the contact points. We fix the atoms at the contact points for the simulations of the graphene membrane with external induced strain. The left and right contacts are separated at a distance L_x from each other and are not allowed to move nor change the effective bond lengths of the underlying carbon atoms. The simulations are preformed for different $L_x = 99.2 - 104.2$ Å which correspond to initial effective bond lengths $a = 1.38 - 1.45$ Å.

The distance L_x is related to the external imposed tension on the graphene membrane. One expects that increasing L_x results in a more stretched membrane and the height fluctuations are reduced. Figure 2 confirms that the height fluctuations $\sqrt{\langle z^2 \rangle - \langle z \rangle^2}$ depend on L_x and therefore also on the strain imposed on the graphene membrane. Interestingly, the height fluctuations are independent on the temperature. In contrast, the width of the effective bond length distribution presents a strong temperature dependence as shown in Figs. 3 a-c.

This finding is related to the Ricci scalar analysis (see Fig. 4 in the main article), where it has been shown that the absolute value of the Ricci scalar has a much stronger temperature dependence than a dependence on L_x. A higher temperature results in a broader width of the effective bond length distribution. This leads to a higher average Ricci scalar and consequently to more curvature in the system. The curvature introduces shear which is responsible for saturation of the current.

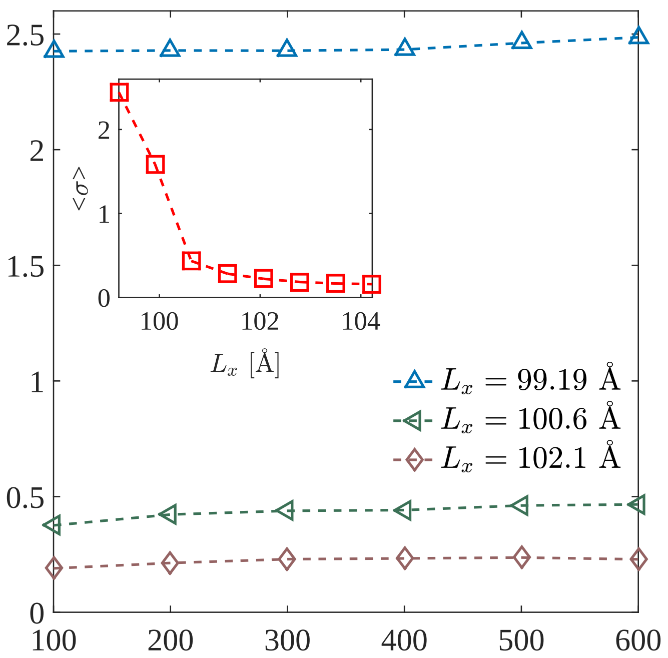

Figure 2. Main figure: Standard deviation of the heights σ as a function of temperature for different $L_x = \{99.2, 100.6, 102.1\}$. Inset: Temperature averaged standard deviation $\langle\sigma\rangle$ for different distances L_x.

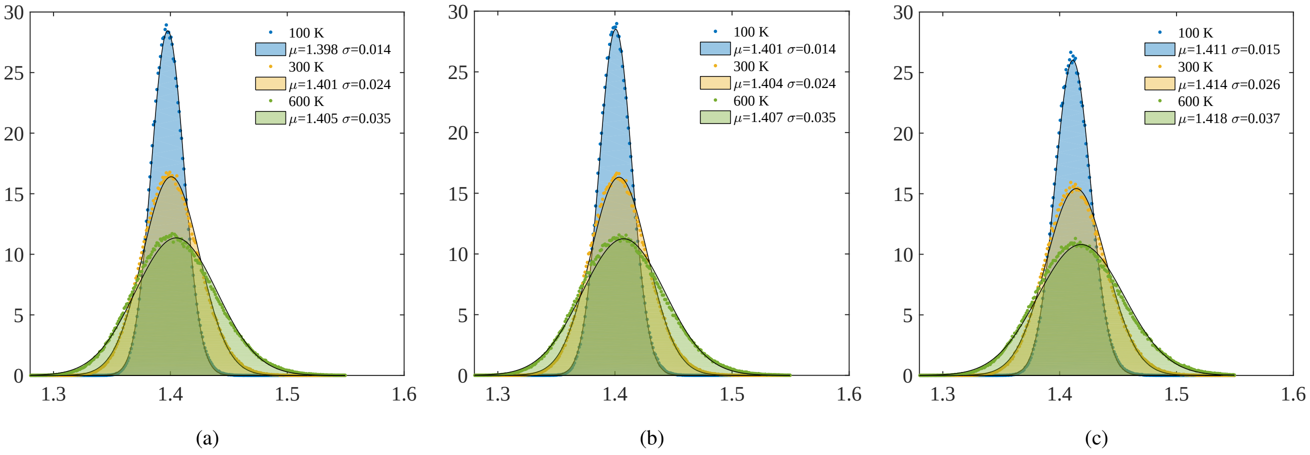

Figure 3. Distribution of the effective bond lengths for $L_x = 99.2$ Å (a), $L_x = 100.6$ Å (b) and $L_x = 102.1$ Å (c). The black line corresponds to a Gauss distribution with mean μ and standard deviation σ.

References

1. Oettinger, D., Mendoza, M. & Herrmann, H. J. Gaussian quadrature and lattice discretization of the fermi-dirac distribution for graphene. *Phys. Rev. E* **88**, 013302 (2013). URL http://link.aps.org/doi/10.1103/PhysRevE.88.013302.
2. Furtmaier, O., Mendoza, M., Karlin, I., Succi, S. & Herrmann, H. J. Rayleigh-bénard instability in graphene. *Phys. Rev. B* **91**, 085401 (2015). URL http://link.aps.org/doi/10.1103/PhysRevB.91.085401.
3. Cercignani, C. & Kremer, G. M. *The Relativistic Boltzmann Equation: Theory and Applications* (Boston; Basel; Berlin: Birkhauser, 2002).
4. Pozrikidis, C. *Fluid dynamics: theory, computation, and numerical simulation* (Springer Science & Business Media, 2009).
5. Ochoa, H., Castro, E. V., Katsnelson, M. I. & Guinea, F. Temperature-dependent resistivity in bilayer graphene due to flexural phonons. *Phys. Rev. B* **83**, 235416 (2011). URL https://link.aps.org/doi/10.1103/PhysRevB.83.235416.
6. Castro, E. V. *et al.* Limits on charge carrier mobility in suspended graphene due to flexural phonons. *Phys. Rev. Lett.* **105**, 266601 (2010). URL http://link.aps.org/doi/10.1103/PhysRevLett.105.266601.
